# Quality assurance review: Intra‐operative evaluation of sentinel lymph nodes in breast cancer

**DOI:** 10.1002/cam4.4264

**Published:** 2021-09-17

**Authors:** Tamadar Aldoheyan, Julianne Klein

**Affiliations:** ^1^ Department of Pathology College of Medicine Rady Faculty of Health Sciences University of Manitoba Winnipeg Manitoba Canada

**Keywords:** axillary lymph nodes dissection, breast cancer, frozen section and touch imprints, intraoperative consultation, sentinel lymph node, SLN

## Abstract

**Background:**

Intraoperative consultation (IOC) of axillary sentinel lymph node (SLN) biopsy continues to play a role in selected breast cancer patients. The reported sensitivity rates for intraoperative SLN evaluation in breast cancer range from 47% to 80%. We study a center where the majority of SLN IOC is performed by imprint cytology, and a protocol was established to limit microscopic examination to three slides for a reporting TAT goal of 30 min.

**Methods:**

Approval to conduct this study was obtained from the REB. A retrospective review was performed on all consecutive SLN cases sent for IOC. Reported IOC assessments of all cases were compared with the final pathology.

**Results:**

Of 164 patients, there were 22 (13%) false negative IOC events, including 15 missed macro‐metastasis and 7 missed micro‐metastasis. The overall sensitivity for touch imprint in detecting SLNs macro‐metastasis was 70.9%. Reporting total turnaround time was on average 3 min longer, whereas sensitivity and specificity were not significantly different in the two protocol periods.

**Conclusion:**

Implementation of an IOC policy for a maximum of three slides for imprint cytology did not result in a significant impact on the sensitivity, specificity, or total turnaround time for SLN in breast cancer patients. False negative IOC events were mainly due to sampling error. Quality review was made difficult by limited documentation related to the gross handling of the specimens at IOC. System factors identified include insufficient space for the IOC report on the pathology requisition, and the lack of clearly communicated expectations for documentation.

## Abstract 2

The sensitivity of touch imprint cytology during intra‐operative consultation in sentinel lymph nodes of breast cancer is not affected by the number of lymph nodes assessed. False negative intra‐operative consultation events were mainly due to sampling error. Quality review was made difficult by limited documentation related to the gross handling of the specimens at intra‐operative consultation. Factors identified include insufficient space for the IOC report on the pathology requisition, and the lack of clearly communicated expectations for documentation.

## 1 INTRODUCTION

Axillary lymph node status is one of the most important prognostic factors in breast carcinoma.^1^ Sentinel lymph node (SLN) biopsy has long been the standard of care for most patients with clinically negative axillary lymph nodes. The Z0011 trial which was completed in 2011 has resulted in fewer Intraoperative consultation (IOC) being performed for SLN status, however intraoperative assessment of axillary sentinel lymph node biopsy continues to play a role in breast cancer patients who do not meet the Z0011 selection criteria.^2^ Intraoperative evaluation of SLNs can be done by frozen section, imprint cytology (IC), smear cytology, or a combination of these methods. Much of the breast surgery in the Province of Manitoba is performed at the largest hospital site in the provincial medical system, where IOC for breast SLN continues to be performed as a cytology‐based service. A standard protocol for IOC was agreed upon by a committee of pathologists and surgeons and implemented in 2018. This protocol limited the microscopic assessment of SLN at IOC to a maximum of three representative slides (or blocks, for other sites that perform frozen sections) for a target reporting time of 30 min or less. If necessary, additional slides could be examined by agreement at the time of surgery, with the understanding that the anticipated TAT would be longer. It was understood that assessment by IOC is not expected to identify isolated tumor cells (deposits less than 0.2 mm) or all micro‐metastasis (deposits 0.2–2.0 mm). The objectives of our study are to assess the reporting accuracy of the IOC assessment by imprint cytology, to compare the reporting accuracy and TAT before and after instituting the IOC protocol, and to assess factors contributing to false negative cases from a quality assurance perspective.

## 2 METHODS

Approval to conduct this study was obtained from the health research ethics board at University of Manitoba.  A retrospective review was performed on all consecutive SLN cases sent for intraoperative consultation between 1st of April 2017 and 31st of January 2019 at the Health Sciences Centre in Manitoba Canada, comparing the groups, before and after implementing the SLN IOC protocol on 1st March 2018. The protocol was developed by a team of pathologists and surgeons who agreed upon a maximum number of slides that would be expected to be examined microscopically by the Pathologist, for a target reporting TAT goal of 30 min. The protocol limited microscopic examination to three imprint slides for cytology, which the pathologist would direct after gross examination with sectioning the submitted lymph nodes at as close to 2 mm intervals as possible. Whereas in the pre‐protocol period, imprint cytology was used as the main method during SLN IOC, however there were no specified reporting time period or any limitation to the number of slides examined. IOC diagnosis of all cases were reviewed and compared with the final pathology results. Cases with false negative IOC (any lymph nodes reported negative at IOC that were reported positive for metastasis at final pathology) were subjected to slide review along with random slides from true positive and true negative IOC cases. The pathologist reviewing the slides was blinded of the IOC reports and diagnoses. All the data were tabulated and processed using SPSS 27.0. Standard computation of sensitivity, specificity, false negative rate, accuracy, positive predictive value, and negative predictive value were calculated, together with their confidence intervals at 95%. Statistical analysis was performed using Wilcoxon rank sum test and a *p* value of less than 0.05 was regarded as significant.

## 3 RESULTS

### 3.1 Turnaround time

The study duration was 22 months that included 11 before and 11 after the implementation of the protocol. A total of 164 cases of SLN IOC were reviewed, with 81 cases before, and 83 cases after the protocol was implemented. Pre‐protocol reporting TAT ranged between 20 and 54 min with a median of 30 min and an average of 32 min. Thirty‐seven out of 81 (45.6%) cases exceeded 30 min for TAT, however, the majority of cases 26/37 (70%) were in the 30–40 min range. In the remainder of cases (8/37) 21.6% were reported at 40–50 min and (3/37 cases) 8.1% exceeded 50 min (Table 1). After implementing the protocol in March 2018, the TAT ranged between 21 and 67 min with a median of 35 min and an average of 35.7 min. Fifty‐eight out of 83 (69.8%) of the cases exceeded the 30 min suggested time, again with most cases 36/58 (62%) in the 30–40 min range. Twenty‐nine percentage of cases were at 40–50 min and 8.6% (5/58 of cases) exceeded 50 min (Table 1). The mean (SD) total TAT in the pre‐protocol group was 32 (7.5) and in the post‐protocol group was 35.7 (9.2). The 3.7 min difference in the average TAT before and after the protocol is statistically significant (*p* value 0.004) and a 95% confidence interval of (1.24–6.21). Similarly, the median (IQR) was 30 (27–35.2) in the pre group and 35 (29.8–40.2) in the post group.

**TABLE 1:** Reporting time‐frames

	Pre‐protocol	Post‐protocol
Total cases	81	83
Range of reporting times	20–54 min	21–67 min
Average reporting TAT	32 min	35.7 min
Median reporting TAT	30 min	35 min
Cases reported in less than 30 min	44 cases	25 cases
Cases reported between 30–40 min	26 cases	36 cases
Cases reported between 40–50 min	8 cases	17 cases
Cases exceeding 50 min	3 cases	5 cases

Abbreviation: TAT, total turn‐around time.

### 3.2 False negatives

**FIGURE 1:**
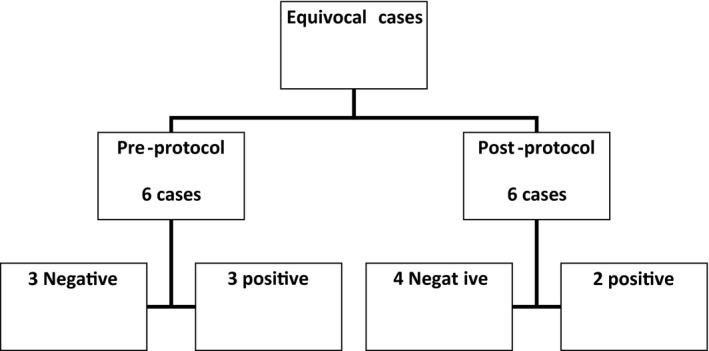
Equivocal cases reported during intraoperative consultation and permanent sections diagnosis (positive and negative)

Out of 164 cases, 22  (13.4%) cases were false negative, including 15 macro‐metastasis and 7 micro‐metastasis. In the period prior to implementing the protocol, 8/81 of cases (9.9%) were false negatives, which included 6 macro‐metastasis and 2 micro‐metastasis. By comparison, in the post‐protocol period 14/83 of cases (16.8%) were false negatives,  including  9 macro‐metastasis and 5 micro‐metastasis. In seven cases reported as negative on IOC, ITC was identified on permanent section, with four and three identified before and after implementation of the protocol, respectively. ITC cases were not considered as missed metastases. There were 12 cases that were reported as equivocal (6 pre‐protocol and 6 post‐protocol) by cytology on IOC. In the pre‐protocol group three cases were considered negative on final review with permanent sections and three were reported as positive for metastasis. In the post‐protocol group four of the equivocal cases were negative and two were positive for lymph node metastasis at final pathology (Figure 1).

### 3.3 Sensitivity and specificity

The sensitivity of identifying macro‐metastasis on TI cytology was 76% in the pre‐protocol cases and 69% in the post‐protocol cases, which was not statistically significant with a *p*‐value of 0.57 for a 95% confidence interval. (Table 2) The overall sensitivity for both the pre‐ and post‐protocol time periods was 70.9%. Specificity was 100% in both time periods, with 19 cases of metastasis identified at IOC pre‐protocol and 20 post‐protocol. The accuracy was 92% and 88% in the pre‐ and post‐protocol, respectively.

**TABLE 2:** Accuracy of intraoperative consultation in identifying lymph node metastasis

	Pre‐protocol	Post‐protocol
Total cases	81	83
True positive	19	20
True negative	54	49
False negative (macro‐metastasis)	6	9
False negative (micro‐metastasis)	2	5
Missed ITCs	4	3
Sensitivity (macro‐metastasis)	76%	69%
Specificity	100%	100%
Accuracy	90%	83%

Abbreviation: ITC, isolated tumor cells

### 3.4 Histologic characteristics of the false negative cases

All 15 patients with macro‐metastasis that were not identified at IOC underwent total mastectomy (nipple‐sparing or skin‐sparing mastectomy) for their primary tumor resection. Of the 15 cases, 13 (86.6%) were invasive ductal carcinoma and 2 (13.3%) were invasive lobular carcinomas. The ductal carcinomas were 30% Nottingham grade 1 (4) 15% grade 2 (2) and 54% grade 3 (7) and both lobular carcinomas were Nottingham grade 2. Forty percentage of the 15 cases with missed macro‐metastasis at IOC had received neoadjuvant treatment and five of them demonstrated therapeutic effects on permanent sections. This is compared to 12/39 of true positive cases (30%) with neoadjuvant treatment that had lymph node metastasis identified at IOC with 10 of them demonstrating therapeutic effects on permanent sections. Of the pre‐protocol cases that were missed, two out of six were metastatic deposits 5 mm or greater in size (33%); compared to seven out of nine 9 (78%) which were 5 mm or greater in the post‐protocol group.

### 3.5 Quality assurance

There were a number of challenges related to documentation that limited the quality assurance review. The number of imprint slides produced and examined at IOC was not consistently documented at the time of the IOC and was not available in the final pathology report or document within the laboratory information system; Based upon slide retrieval, the average number of slides examined was estimated to be 2.3 in the pre‐protocol period, and 2.6 in the post‐protocol period. Only four cases out of 15 reported the number of lymph nodes received and examined at IOC. The number of lymph nodes reported in the final pathology report ranged from 1 to 7 with an average of 2.5 lymph nodes. Of the 15 cases with missed macro‐metastasis, only one case recorded size of the lymph nodes submitted for IOC. Based on measurements at the time of final gross description, the lymph nodes ranged in size from 2.5 mm to 20 mm with an average of 6.3 mm. Documentation that the lymph nodes were cross sectioned at 2–3 mm intervals at the time of IOC was in the reports in only 6/15 of cases. Correlating the lymph node size estimates documented in the final gross description and the number of sections per block suggests that only three of the 15 cases may have been sectioned at 2–3 mm intervals at the time of IOC. Out of the 15 cases with missed macro‐metastasis, four cases included the number of lymph nodes examined during IOC and only one case recorded size of the lymph nodes submitted for IOC. In only five out of the 15 cases did the final report document the reason for the discrepancy between the IOC and the final report.

### 3.6 Axillary lymph node dissection (ALND)

Of the 15 cases that were falsely negative for macro‐metastasis at IOC, two patients had ALND performed. One patient had an “equivocal” IOC cytology result, and the Surgeon opted to perform the ALND at the time of the primary operation. The other patient's result had been reported as negative, and this patient underwent a subsequent procedure (Table 3 and Table 4). Of the 39 true positive cases, 35 (89.7%) of cases underwent ALND at the initial procedure.

**TABLE 3:** Missed macro‐metastasis cases–pre‐protocol

Case No.	No. of LNs examined	No. of LNs submitted	No. of slides examined at IOC	IOC diagnosis	No. of positive LNs at permanent/total	Size of metastasis at permanent	ENE	Histologic type and procedure	Size of tumor and pT stage	Tumor grade	ER/PR HER2 status	NAT	Treatment effect	Documentation of reasons behind missed macro‐metastasis	Completion of ALND
1	ND	ND	1	Equivocal	1/1	0.3 cm	No	IDC, mastectomy	3.1 cm pT2	G3	+ER/PR +HER2	No	N/A	ND	No
2	ND	ND	2	Negative	1/1	0.4 cm	Yes	ILC, mastectomy	8 cm pT3	G2	+ER/PR −HER2	No	N/A	ND	No
3	ND	ND	2	Equivocal	2/2	0.6 cm	Yes	IDC, mastectomy	1.2 cm ypT1c	G1	+ER/PR −HER2	Yes	Yes	ND	Yes, during the same procedure
4	ND	ND	2	Equivocal	1/2	0.6 cm	No	IDC, mastectomy	1.9 cm pT1c	G3	Triple negative	No	N/A	ND	No
5	2	2	2	Negative	2/2	0.4 cm	No	IDC, mastectomy	4.9 cm pT2 (m)	G3	+ER/PR +HER2	No	N/A	Review of imprints were negative	No
6	ND	ND	3	Negative	3/7	0.3 cm	No	IDC, mastectomy	1.3 cm ypT1c	G1	+ER/PR HER2 equivocal	Yes	Yes	ND	No

Abbreviations: ALND, Axillary lymph node dissection; ENE, extranodal extension; G, grade; IDC, invasive ductal carcinoma; ILC, invasive lobular carcinoma; IOC, intraoperative consultation; LNs, lymph nodes; N/A, not applicable; NAT, Neoadjuvant treatment; No., Number; ND, Not documented; pT, pathologic tumor stage.

**TABLE 4:** Missed macro‐metastasis cases–post‐protocol

Case No.	No. of LNs examined	No. of LNs submitted	No. of slides examined at IOC	IOC diagnosis	No. of positive LNs at permanent/total	Size of metastasis at permanent	ENE	Histologic type and procedure	Size of tumor and pT stage	Tumor grade	ER/PR HER2 status	NAT	Treatment effect	Documentation of reasons behind missed macro‐metastasis	Completion of ALND
1	6	3	3	Negative	1/3	0.5 cm	No	ILC, mastectomy	5.3 cm pT3	G2	+ER/PR −HER2	No	N/A	ND	No
2	ND	ND	3	Equivocal	1/3	0.7 cm	Yes	IDC, mastectomy	1.6 cm ypT1c (m)	G3	Triple negative	Yes	Yes	ND	No
3	ND	ND	3	Negative	½	0.5 cm	No	IDC, mastectomy	2.8 cm pT2 (m)	G2	+ER −PR −HER2	No	N/A	ND	Yes, as a second procedure
4	ND	ND	3	Negative	1/3	0.3 cm	No	IDC, mastectomy	0.4 cm ypT1a	G3	Triple negative	Yes	No	ND	No
5	5	5	5	Negative	3/6 (2 micro and 1 macro)	1.0 cm	No	IDC, mastectomy	4 cm pT2 (m)	G3	+ER −PR −HER2	No	N/A	Review of imprints were negative	No
6	ND	ND	2	Negative	1/2	0.8 cm	No	IDC, mastectomy	1.5 cm pT1c	G2	+ER Equivocal PR and HER2	No	N/A	Review of imprints were negative	No
7	3	3	3	Negative	3/3 (2 micro and 1 macro)	0.23 cm	No	IDC, mastectomy	2.8 cm pT2	G3	−ER/PR +HER2	No	N/A	Review of imprints were negative	No
8	ND	ND	2	Negative	2/2	1.5 cm	ND	IDC, mastectomy	14.5 cm ypT3	G1	+ER/PR −HER2	Yes	Yes	Review of imprints were negative	No
9	ND	ND	1	Equivocal	1/1	0.5 cm	No	IDC, mastectomy	3.5 cm ypT2 (m)	G1	+ER/PR −HER2	Yes	Yes	ND	No

Abbreviations: ALND, Axillary lymph node dissection; ENE, extranodal extension; G, grade; IDC, invasive ductal carcinoma; ILC, invasive lobular carcinoma; IOC, intraoperative consultation; LNs, lymph nodes; N/A, not applicable; NAT, Neoadjuvant treatment; No., Number; ND, Not documented; pT, pathologic tumor stage.

## 4 DISCUSSION

The average reporting TAT in the post‐protocol period unexpectedly increased by 3.7 min, which while statistically significant, is not considered clinically significant. The average number of imprint slides examined increased from 2.3 to 2.6 per case, which likely contributed to the additional reporting time. This observation was interested in that we assumed the number of slides examined and reporting time would decrease but it did not. Prior to the initiation of the protocol, we did not have comprehensive data on the overall TAT or the numbers of slides examined. In retrospect, the protocol was prompted by complaints that were likely related to a number of cases with TAT outliers in the pre‐protocol period. In addition, there was a pre‐protocol perception by pathologists that large numbers of lymph nodes were being submitted for IOC. A significant proportion of cases (54% in the pre‐protocol period and 30% in the post‐ protocol period) were reported in less than 30 min, 26/81 (32%) of cases in the pre‐protocol period and 36/83 of cases (43%) in the post‐protocol period were reported by 40 min. The number of cases that exceeded 40 min was unexpectedly greater after implementation of the “3 slide” microscopic examination protocol. Factors that contribute to the reporting TAT could not be specifically reviewed as part of the study due to a lack of documentation. There are many potential factors that could contribute to reported time that could be investigated. This includes time to specimen delivery to pathology, multipart specimens with cumulative reporting times, the process of pre‐pathologist cytotechnologist screening of the slides, and time to delivery of the slides to the pathologist.

We report an overall sensitivity and specificity for IOC IC in identifying macro‐metastasis and micro‐metastasis in SLNs is of 64% and 100%, respectively. Which is within the range of sensitivity published in the literature, a meta‐analysis of 31 studies that evaluated intraoperative IC for SLNs by Tew et al.^3^ indicated that the pooled sensitivity of IC was 63%, and its specificity was 99%. In a prospective study of IOC IC, the sensitivity and specificity were 50% and 100%, respectively.^4^ In a multicentric and retrospective study on more than 2000 SLN the sensitivity of IC was 32.4% and 99.2% specific.^5^ The difference in macro‐metastasis sensitivity in the two study periods (76% pre‐protocol, and 69% post‐protocol) was not statistically significant. This corresponds to a false negative rate for macro‐metastasis of 29% (24% and 31% pre‐ and post‐protocol, respectively). Memar B. et al. reported a sensitivity of TI for SLN IOC of 80%.^6^ Mori et al.^7^performed a study on 138 patients and reported 47.1% and 88.2% sensitivity for TI cytology and FS, respectively. Since the majority of cases are done by cytology by convention in the department where the study was performed, comparison with FS was not part of our study.

During both study periods more cases of missed metastasis were macro‐metastasis (15 cases) than micro‐metastasis (7 cases). A retrospective study of 1227 patients evaluating TI cytology on axillary SLN showed a higher false negative rate in macro‐metastasis (49 cases) compared to micro‐metastasis (39 cases).^8^ Even with the same method of intraoperative assessment of SLN, the sensitivity and false negative rates will be dependent on numerous variables, which render published series incomparable. For example, some serially sectioned at 2 mm intervals if they were 5 mm or more in diameter; or bisected if they were less than 5 mm and touch‐imprint both sides of each slice,^4^, ^9^ whereas others bisect nodes along the long axis and two imprints made from each SLN,^8^ serially section in 2–3 mm slices,^10^ or use a combination of methods.^11^ Serial sectioning invariably increases the sensitivity of touch preparation or frozen section over simply examining the cut sides of the bisected node.^12^ In addition, the rate of nodal positivity in published series is dependent on whether immunohistochemistry (IHC) is used or not.^13^, ^14^, ^15^ That being said it is clear from the published series that whatever method is used to evaluate the sentinel node during surgery, more comprehensive examination of the lymph nodes results in a higher yield of identified metastasis.^16^

Missed LN metastasis at the time of IOC can be the result of specimen sampling (involved lymph node not sampled, or involved area of the lymph node not sampled for microscopic examination) or interpretation (carcinoma cells present on the slide but too few or closely resembling lymphocytes or histiocytes thus not identified by the pathologist). In our study five out of 15 false negative cases documented the reason for the discrepancy with the IOC in the final report, which in all cases were due to specimen sampling. Review of the IOC imprint cytology slides confirmed that all were negative. For cases with more than one lymph node assessed at IOC, the imprint slides could not be reliably correlated to a specific LN on permanent sections. For example, one case had six lymph nodes submitted and three imprint slides performed, and on final pathology one of the six LN was positive for metastatic carcinoma. Since all lymph nodes were placed back into their original container at IOC with no designation as to which nodes correspond to the imprint slides, it was impossible to identify if the positive lymph node had been imprinted at IOC. There was insufficient documentation to determine whether the pathologist had personally grossly examined and sectioned all lymph nodes submitted at IOC, or whether this had been performed by a pathologist assistant.

**FIGURE 2:**
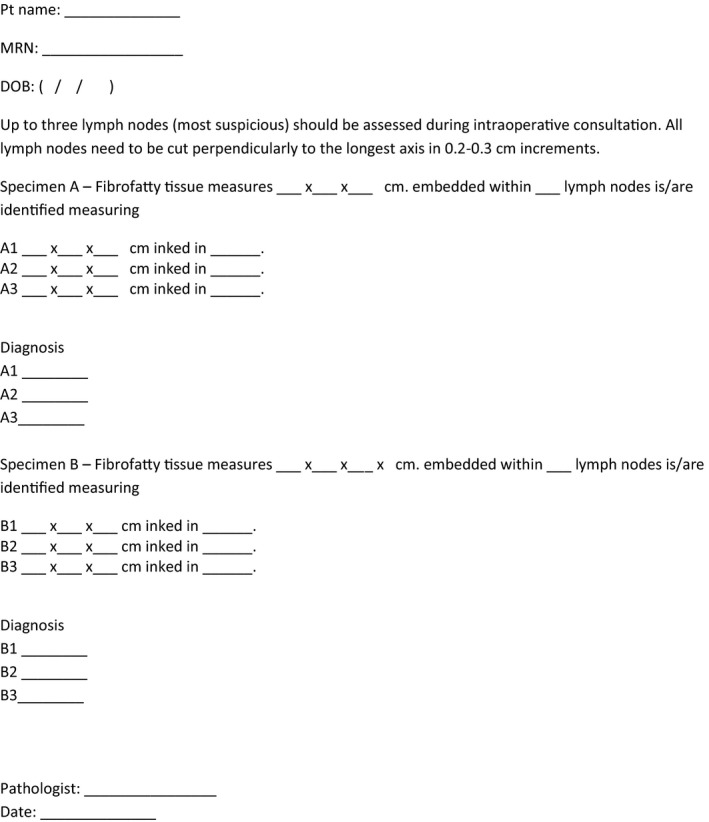
Suggested worksheet for proper lymph node handling during intraoperative consultations

Many centers currently perform IOC by FS, which is considered by some to be more accurate than IC.^17^ Others such as Menes et al.^18^ have indicated that the sensitivity of touch preparation is comparable to that of FS and since touch preparation is more rapid and less expensive, uses less tissue, and does not create freezing artifacts, they concluded that touch preparation is the best available method for intraoperative evaluation of SLN. Newer studies also showed that IC is an effective and quick method for detecting macro‐metastasis in breast SLN.^19^, ^20^ One other limitation is that the documentation at the time of IOC was limited, and it was therefore impossible to accurately assess the adequacy of gross assessment as well as specimen sampling. The main finding in our study indicate that a maximum of three slides or three blocks for microscopic examination did not impact the sensitivity of SLN IOC. Since most of the cases that were missed were macro‐metastases, to reduce the false negative cases, there is a need of focusing on grossing of the specimen and selecting sections for microscopic assessment. Quality assessment was hindered by incomplete documentation of the gross handling of the specimen. We felt that this was most likely related to insufficient space on the requisition, and a lack of defined expectations for what documentation was required. In order to improve the system, we plan to institute a work‐sheet to facilitate ease of recording of information during the gross assessment and handling of the specimen. (Figure 2). Documentation of serial cross sections per SLN protocol, and the presence of grossly suspicious areas for targeted microscopic examination (by imprints or frozen section) on specifically labelled slides will be recommended. The use of colored inks will be considered, to correlate the gross specimen to the IOC slides examined when multiple lymph nodes are present in a single container.

In conclusion, following the institution of a pathology protocol for IOC on SLN in breast cancer patients, the average reporting TAT for IOC by cytology touch imprints in our study increased by about 3 min, and the average number of slides examined increased from 2.3 to 2.6. The majority of cases were reported by 40 min. The overall sensitivity in our study is comparable to that which is reported in the literature; however, a greater number of the missed metastasis at IOC was macro‐metastasis than micro‐metastasis. This was an unexpected finding suggesting that a focus on gross assessment and specimen sampling could provide an opportunity to decrease the false negative rate. Quality assurance review was limited by a lack of standardized documentation. We plan to incorporate use of a worksheet to support documentation of specimen handling as a quality initiative resulting from this study.

## CONFLICT OF INTEREST

The authors whose names are listed immediately below certify that they have NO affiliations with or involvement in any organization or entity with any financial interest in the subject matter or materials discussed in this manuscript.

## ETHICS STATEMENT

The authors are accountable for all aspects of the work in ensuring that questions related to the accuracy or integrity of any part of the work are appropriately investigated and resolved.

## Data Availability

The data that support the findings of this study are available from the corresponding author upon reasonable request.
